# Retraction Note: Secretome of endothelial progenitor cells from stroke patients promotes endothelial barrier tightness and protects against hypoxia-induced vascular leakage

**DOI:** 10.1186/s13287-024-03667-7

**Published:** 2024-02-27

**Authors:** Rodrigo Azevedo Loiola, Miguel García-Gabilondo, Alba Grayston, Paulina Bugno, Agnieszka Kowalska, Sophie Duban-Deweer, Eleonora Rizzi, Johan Hachani, Yasuteru Sano, Fumitaka Shimizu, Takashi Kanda, Caroline Mysiorek, Maciej Piotr Mazurek, Anna Rosell, Fabien Gosselet

**Affiliations:** 1 UR 2465, Blood-Brain Barrier Laboratory (LBHE), Univ. Artois, Lens, 62300 France; 2 Neurovascular Research Laboratory, Vall d’Hebron Institut de Recerca, Universitat Autònoma de Barcelona, Barcelona, 08035 Catalonia Spain; 3 Pure Biologics S.A., Wroclaw, 54–427 Duńska 11 Poland; 4 Department of Neurology and Clinical Neuroscience, Graduate School of Medicine, Yasuteru Sano, Yamaguchi University, Ube, Japan; 5 Laboratory of the Blood-Brain Barrier, Sciences Faculty Jean Perrin, Artois University, Lens, France

**Stem Cell Research & Therapy (2021) 12:552**

10.1186/s13287-021-02608-y

The authors have retracted this article. After the publication of this article, the authors carried out a voluntary and exhaustive investigation of the endothelial progenitor cells (EPC) cryovials used in this study. This investigation was performed to verify the authenticity of human cell lines as a quality control of stored biological material by Short Tandem Repeat (STR) genotyping. Unfortunately, the results of this investigation revealed that the cells used in this study were verified as hCMEC/D3 with > 95% probability using the Cellosaurus STR Similarity Search Tool, which falls within the accepted verification percentage, and not as primary cells, as expected for the endothelial progenitor cells. These results were also verified by a second external laboratory of analysis and notified to participating laboratories and to the Research Integrity Committee of the cell source laboratory.

The authors apologize to the community for this unexpected finding, which has led to the retraction of this article.

All authors agree with this retraction.

